# Serum antibody response to Human papillomavirus (HPV) infections detected by a novel ELISA technique based on denatured recombinant HPV16 L1, L2, E4, E6 and E7 proteins

**DOI:** 10.1186/1750-9378-1-6

**Published:** 2006-11-08

**Authors:** Paola Di Bonito, Felicia Grasso, Stefania Mochi, Luisa Accardi, Maria Gabriella Donà, Margherita Branca, Silvano Costa, Luciano Mariani, Alberto Agarossi, Marco Ciotti, Kari Syrjänen, Colomba Giorgi

**Affiliations:** 1 Department of Infectious, Parasitic and Immunomediated Diseases, Istituto Superiore di Sanità. (ISS), Rome, Italy; 2 Unità Citoistopatologica, Centro Nazionale di Epidemiologia, Sorveglianza e Promozione della Salute, ISS, Rome, Italy; 3 Dipartimento di Ginecologia e Ostetricia, Azienda Ospedaliera S. Orsola Malpighi, Bologna, Italy; 4 Ginecologia e Ostetricia, IFO, Istituto Regina Elena, Rome, Italy; 5 Clinica Ostetrica e Ginecologica, Istituto Scienze Biomediche, Ospedale Luigi Sacco, Milano, Italy; 6 Laboratory of Clinical Microbiology and Virology, University Hospital "Policlinico Tor Vergata", Rome, Italy; 7 Department of Oncology and Radiotherapy, Turku University Hospital, Turku, Finland; 8 on behalf of the HPV-PathogenISS group #

## Abstract

**Background:**

Human papillomaviruses (HPVs) are the primary etiological agents of cervical cancer and are also involved in the development of other tumours (skin, head and neck). Serological survey of the HPV infections is important to better elucidate their natural history and to disclose antigen determinants useful for vaccine development. At present, the analysis of the HPV-specific antibodies has not diagnostic value for the viral infections, and new approaches are needed to correlate the antibody response to the disease outcome. The aim of this study is to develop a novel ELISA, based on five denatured recombinant HPV16 proteins, to be used for detection HPV-specific antibodies.

**Methods:**

The HPV16 L1, L2, E4, E6 and E7 genes were cloned in a prokaryotic expression vector and expressed as histidine-tagged proteins. These proteins, in a denatured form, were used in ELISA as coating antigens. Human sera were collected from women with abnormal PAP smear enrolled during an ongoing multicenter HPV-PathogenISS study in Italy, assessing the HPV-related pathogenetic mechanisms of progression of cervical cancer precursor lesions. Negative human sera were collected from patients affected by other infectious agents. All the HPV-positive sera were also subjected to an avidity test to assess the binding strength in the antigen-antibody complexes.

**Results:**

Most of the sera showed a positive reactivity to the denatured HPV16 proteins: 82% of the sera from HPV16 infected women and 89% of the sera from women infected by other HPV genotypes recognised at least one of the HPV16 proteins. The percentages of samples showing reactivity to L1, L2 and E7 were similar, but only a few serum samples reacted to E6 and E4. Most sera bound the antigens with medium and high avidity index, suggesting specific antigen-antibody reactions.

**Conclusion:**

This novel ELISA, based on multiple denatured HPV16 antigens, is able to detect antibodies in women infected by HPV16 and it is not genotype-specific, as it detects antibodies also in women infected by other genital HPVs. The assay is easy to perform and has low cost, making it suitable for monitoring the natural history of HPV infections as well as for detecting pre-existing HPV antibodies in women who receive VLP-based HPV vaccination.

## Background

Human Papillomaviruses (HPVs) infect cutaneous, genital, and respiratory epithelia in tissue-specific manner. Papillomaviruses are non-enveloped viruses with a double-strand DNA genome of 8 Kb. The genome is encapsidated in an icosahedral structure of 55 nm-diameter, composed of the L1 and L2 proteins, which are the major and minor capsid protein, respectively. The HPV16 genome codes for several non-structural proteins: E1, E2, E4, E5, E6 and E7. E1 and E2 are essential in viral transcription and replication, E4 binds cytokeratins and is involved in modification of the cytoskeleton network; E5 affects cellular receptors of growth factors, whereas E6 and E7 are the major transforming proteins [1].

HPV infections are widespread in the general population, and viral infection is closely associated to both benign and malignant lesions [2,3]. Studies performed by several groups have recently established that only few of the over 30 genital HPV genotypes described, are important risk factors for developing high-grade of cervical intraepithelial neoplasia (CIN3) and cervical cancer (CC), with a high prevalence of genotype 16 [4,5]. Serological studies are important to understand the natural history of HPV infections, and during the past 15 years, efforts have been made to develop reliable genotype-specific serological assays. Most of the sero-epidemiological studies have focused on confirming the relationship between the presence of HPV antibodies and the detection of anogenital cancers or their precursors. The majority of these studies have used either virus like particles (VLPs) or E6/E7 oncoprotein-based serological assays, while other HPV proteins have been used as antigens less frequently [2,6,7].

The main conclusion from these sero-epidemiological studies is that the antibody response to HPV16 proteins does not invariably occur during a natural HPV infection. For example a humoral immune response to VLPs is induced in about half of the women with normal cytology and HPV DNA presence in their cervical epithelium. The VLP-specific antibodies are neutralizing and genotype-specific and have been crucial for the development of preventive HPV vaccines [8,9]. An anti-HPV16 E6/E7 response has been predominantly found in patients with advanced CC, but it has also been found in control patients, making doubtful the use of serology in cancer prediction [10]. However prospective cohort studies using a widespread panel of viral antigens are urgently needed to improve our understanding on HPV seroconversion in patients with and without cervical lesions, as well as to study the dynamics of antigen-specific HPV antibodies in relation to the clinical outcome of the viral infection.

This paper reports the development of an in-house ELISA system, based on five HPV16 proteins expressed in *Escherichia coli *(*E. coli*). The assay uses the recombinant viral L1 and L2 capsid proteins, E6 and E7 oncoproteins, and non-structural E4 protein, all in denatured form. The assay has been tested to monitor the antibody response in women enrolled in the HPV-PathogenISS cohort to study the HPV-related pathogenetic mechanisms of CIN progression in HIV-negative and HIV-positive women [11].

## Results and discussion

### Analysis of the HPV genotypes

Before testing the 99 sera for the presence of HPV antibodies, DNA samples from cervical smears of the same patients were analysed for the presence of HPV DNA, followed by virus genotyping. HPV sequences were found in all the samples, but the identification of a specific HPV genotype was possible only in 66 samples. In the present study these 66 cases were considered (Table 2). Among the 18 genotypes detected, 12 (66.7%) were "high risk" HPV (HR-HPV), 1 (genotype 90) was of "indeterminate risk" and 5 were "low risk" HPV (LR-HPV) according to the epidemiological classification of Munoz and co-workers [12]. The most common HPV genotypes in descending order of frequency were: HPV16 (33.3%), HPV35 (9.1%), HPV31 (7.5%), HPV6-18-52-66 (6%). HPV16 was the most prevalent genotype; half of the infections were associated to the HR 16, 35, 31 genotypes, whereas HPV18 was found in only 6% of the women. The prevalence of HPV16 is similar to that previously reported in Italy [13-17] whereas that of the other genotypes is very different, probably due to the small number of samples analysed.

**Table 1:** HPV primers used in PCR amplification of the HPV 16 genes.

**Primer**	**DNA sequence**	**Gene target**	**Nucleotide positions in NCBI no **NC_001526
L1_Sfor	5-GCCGTCGAC**ATG**TCTCTTTGGCTGCCTAGTAGGGCCA-3'	Late protein L1	5637–5663
L1_Srev	5'- GCCGTCGAC**GAT**TTGTAGTAAAAATTTGCGTCC-3'	Late protein L1	7027–7050
L2_Bfor	5'-GCGCGGATCC**ATG**cgacacaaacgttctgcaa -3'	Late protein L2	4235–4256
L2_Hrev	5'-GCGCAAGCTT**CTA**GGCAGCCAAAGAGACATC -3'	Late protein L2	5636–5656
E6_Bfor	5'-GCGCGGATCC**ATG**CACCAAAAGAGAACTGC3'	Early protein E6	83–102
E6_Hrev	5'-GCGCAAGCTT**TTA**CAGCTGGGTTTCTCTACGTG -3'	Early protein E6	537–559
E4_Bfor	5'-GCGCGGATCC**ATG**TATTATGTCCTACATCTGTGTTT -3'	Early protein E4	3332–3357
E4_Hrev	5'-GCGCAAGCTT**CTA**TGGGTGTAGTGTTACTATTA-3'	Early protein E4	3597–3619

The start and stop codons are **bolded **while the restriction sites (Sal I, Bam HI, and Hind III) are underlined

**Table 2:** HPV Genotypes and Serostatus.

**Serum n°**	**HPV**	**Sero-reactivity to HPV16 Proteins**				
		
		**L1**	**L2**	**E4**	**E6**	**E7**
1	16	P	P	P	P	P
2	16	P	P			
3	16	P	P	P	P	P
4	16					P
5	16					
6	16		P			P
7	16	P	P	P	P	P
8	16	P	P		P	
9	16	P	P			P
10	16		P			
11	16					P
12	16	P	P			P
13	16	P				P
14	16					
15	16		P			
16	16		P			
17	16	P				
18	16		P			
19	16	P	P			P
20	16					
21	16	P	P	P	P	P
22	16					
23	35		P			
24	35	P	P	P	P	P
25	35					
26	35					P
27	35					P
28	35	P	P			P
29	31					
30	31	P				
31	31	P	P		P	
32	31	P	P	P	P	P
33	31					P
34	6			P		
35	6	P	P	P	P	P
36	6		P		P	
37	6		P			P
38	52	P	P		P	P
39	52					P
40	52					P
41	52	P				P
42	18					P
43	18		P		P	P
44	18					
45	18		P			
46	66	P				P
47	66				P	P
48	66	P	P			P
49	66					
50	53	P	P		P	P
51	53					P
52	53		P			P
53	51	P		P		P
54	51		P			
55	56					
56	56	P				
57	81	P	P	P		P
58	81		P	P	P	P
59	6b	P	P	P	P	P
60	11	P	P	P	P	P
61	39	P				P
62	42	P	P			
63	54				P	P
64	58	P	P	P		P
65	73		P	P	P	P
66	90	P	P	P	P	P

P, positive test; empty cells represent negative tests.

### Analysis of specific antibodies in HPV patients

**Figure 1:**
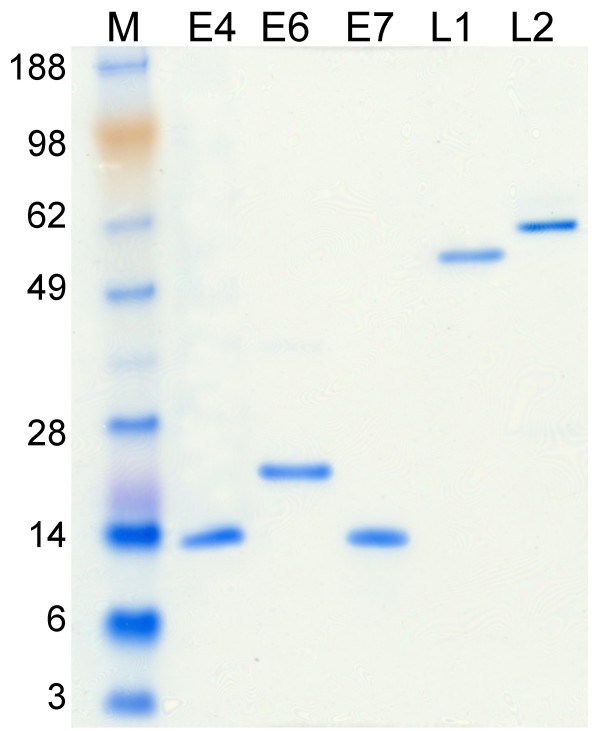
**Analysis of purified proteins by SDS-PAGE**. The purified HPV16 proteins E4, E6, E7, L1 and L2 were run on a polyacrilamide gel electrophoresis and stained by Coomassie blue. Each protein is indicated on the top of the corresponding lane. The weight of the molecular mass markers (lane M) is indicated on the left of the figure.

To study the HPV-specific antibody response, several ELISAs were set up in parallel using the recombinant HPV16 L1, L2, E4, E6 and E7 proteins as coating antigens. Figure 1 shows the SDS-PAGE analysis of the proteins expressed in *E. coli *and purified. The protein pattern shows bands of expected molecular mass; the level of protein purity is over 95%. The proteins were inoculated into mice to generate specific hyper-immune sera. All the viral proteins showed high immunogenicity, inducing specific antibodies reacting with the corresponding antigen in ELISA, Immunoprecipitation and in Western blotting experiments (data not shown).

**Figure 2:**
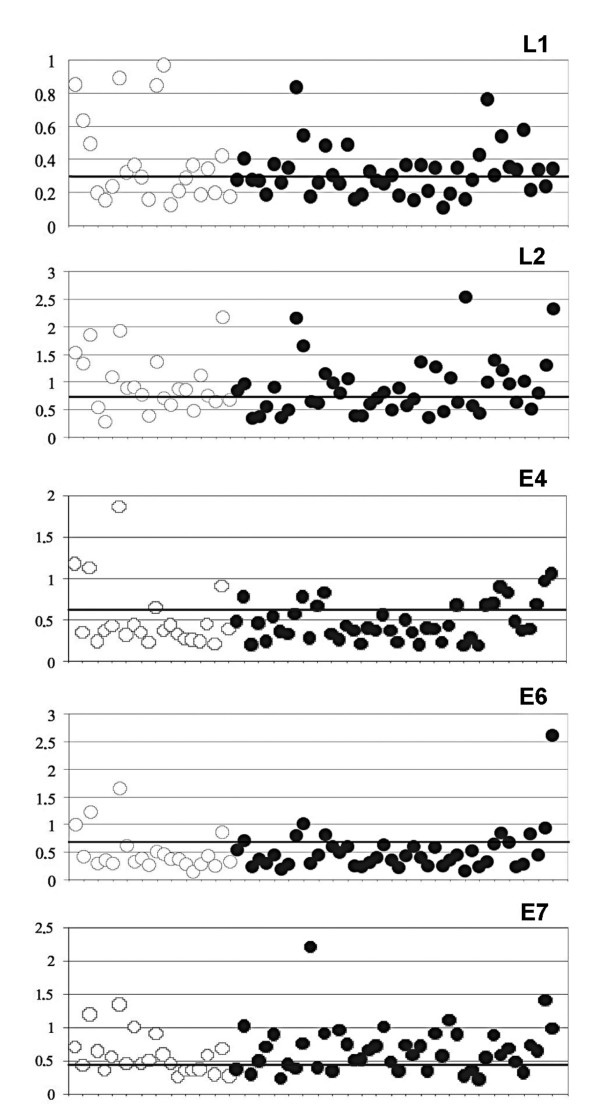
**ELISA based on HPV16 L1, L2, E4, E6 and E7 coating antigens**. Detection of specific immunoglobulins to the HPV16 proteins in sera of women infected by HPV 16 (empty dots) or by other HPVs (black dots). The protein used as coating antigen is indicated on each panel. In abscissa is reported the number of each serum as listed in Tab.2; in ordinate is reported the optical density of each reaction.

In the attempt to identify HPV specific and pan-reactive antibodies, we set up an ELISA using the HPV16 recombinant proteins in denatured form, with carefully determined cut-off values (see below). Among the 66 sera examined, 22 derived from women infected by HPV16, while 44 cases from women infected by other HPV genotypes. The results of a representative multiple HPV-ELISA are shown in Figure 2. The results of seroreactivity to the single HPV16 proteins, related to the HPV genotype data, are listed in Table 2. The highest number of positive reactivity was observed against L1, L2 and E7, while only a few sera had antibodies to E6 and E4. Only few sera were able to react with all the HPV16 antigens and were collected from women infected by HPV16, 31, 35, 6, 6b, 11 and 90 genotypes. These genotypes are correlated to some extent [18]; HPV31 and 35 belong to the same viral species as HPV16; HPV6, 6b and 11 belong to species related to HPV16, whereas HPV90 belongs to a species only distantly related to HPV16. The reactivity to E6 was always associated with reactivity to other viral antigens as well. Some sera reacted only to one antigen, which was L1, L2, E4 or E7. Among the 22 sera from HPV16-positive women, 18 (82%) were positive at least to 1 HPV16 protein, while the rest (18%) were negative to all proteins. The negative result indicates a complete lack of antibody response in the patient, although we cannot exclude that the presence of mutations in the genome of infecting virus could determine the lack of antibody reactivity.

**Table 3:** Avidity Index in the two serum groups.

**HPV16 Group:**	**L1**	**L2**	**E4**	**E6**	**E7**	**Tot**
High AI > 40	10	14	6		10	40
Low AI < 40	1			5	1	7
						
**Other HPVs Group:**						
High AI > 40	21	22	11	3	28	85
Low AI < 40			2	14	2	18

Among the 44 sera of women infected by other HPV genotypes, 39 (89%) reacted at least to one of the five HPV16 proteins, and only 5 (11%) did not react at all. Of all the 66 sera analysed, 57 (86%) were reactive at least to one HPV16 protein, and 9 sera (14%) were entirely negative. The reproducibility of the assay was high as indicated by the inter-assay Coefficient of Variation (CV) of about 5%. The response of the different sera to the same antigen is variable and the differences might reflect the different stage of viral infections.

**Figure 3:**
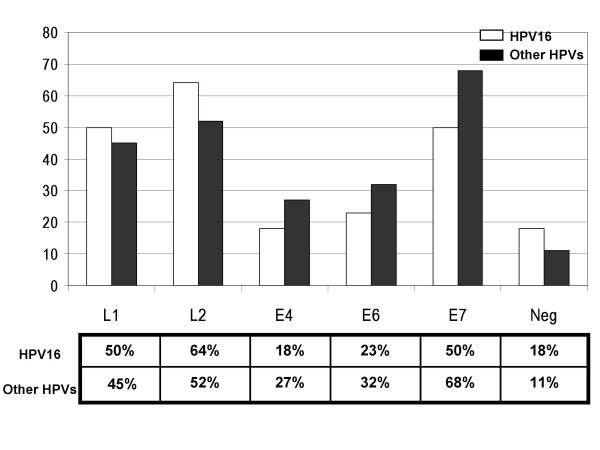
**Comparison of the percentages of sero-reactivity**. The percentages of sera reacting to the HPV16 L1, L2, E4, E6 and E7 proteins are shown; the percentages of the HPV16 serum group are in white bars and those the other HPVs serum group are in black bars. The percentages of negative sera in the two groups (Neg) are also given.

Figure 3 shows the percentage of sera positive to each protein in the two groups of sera: 1) HPV16 and 2) other HPVs. In the first group, the percentage of sera reactive to L2 (64%) is higher than that reactive to L1 (50%) or E7 (50%). In the second group the percentage of sera reacting to E7 (68%) is higher than that reactive to L2 (52%) or L1(45%). Of note the percentage of sera reacting to E7 is higher in the group infected by other HPVs rather than in the HPV16 group. These data suggest that linear and immunogenic epitopes are present in these proteins; these linear epitopes are shared by several genotypes, and account for the cross-reactivity among the sera of women infected by different HPV genotypes.

The percentage of negative sera, shown in Figure 3, is 18% and 11% respectively in the HPV16 and in the other HPVs group. This finding could suggest that HPV16 is less immunogenic than the other HPV genotypes, at least as far as it concerns the humoral response against linear epitopes revealed by our immunoassay. A different immunogenicity of HPV genotypes could account for the markedly different prevalence of HPV genotypes in different geographic regions [5]. Taken together, these results suggest that our multiple ELISA system, based on denatured HPV16 proteins, could be used for detection of HPV-specific antibodies in women infected by different viral genotypes, although a larger number of sera needs to be analysed for its standardization.

### Avidity of the HPV antibodies

The Avidity Index (AI) of specific immunoglobulins is a useful parameter for diagnosis of primary or past infection in a large number of viral diseases [19]. It is well known that after an infection or an immunization, the titer of serum specific immunoglobulin as well as the antibody affinity to the antigen, increase [20]. Several studies have shown that early after an infection serum antibodies have an AI lower than 40%; during the infection the AI values progressively increase with time after infection, achieving an AI higher than 60% [21-24]. This parameter is measured by assaying the resistance of antigen-antibodies complexes to denaturing substances [25]. To evaluate the specificity of each antigen-antibody complex in this HPV-ELISA, we determined the AI in 32 sera positive to L1, 36 to L2, 19 to E4, 22 to E6 and 41 to E7. The HPV-specific mouse hyper-immune sera and the commercial monoclonal antibodies described in Methods were used as a control of the assay. The AI values of the hyper-immune sera were: 44% (anti-L1), 50% (anti-L2), 78% (anti-E4), 26% (anti-E6) 46% (anti-E7). The anti-L1 and anti-E7 mAb AIs were 3% and 5% respectively; the anti-His mAb AI was 20%. The AI results of the human sera are summarised in Table 3. Most samples showed an AI higher than 40% against L1, L2, E7 and E4, but not E6. In the HPV16 group, 40 samples showed an AI higher than 40%, and the AI was below 40% in only 7 samples. In the second group (other HPVs), 85 samples showed an AI higher than 40%, and 18 samples had AI lower than 40%. Most serum samples positive to E6 showed a low AI. Summarizing, four out of five HPV16 proteins tested as antigens seem to be able to bind antibodies with high avidity (AI >40%). These results suggest that L1, L2, E4 and E7 in denatured form could be considered as valid antigens to be used in immunoassays. This is not the case of the HPV16 E6 protein, the linear epitopes of which are recognised with weak avidity by the antibodies. The presence of immunoglobulins with high avidity in the HPV sera might suggest that most of the women with abnormal PAP test enrolled in this cohort have been infected for a prolonged time. This issue merits further investigation, because comprehensive data on the appearance of HPV-specific antibodies to each viral protein and on the variation of their avidity have not been reported before.

## Conclusion

In this study, an in-house ELISA based on the recombinant HPV16 L1, L2, E4, E6 and E7 proteins in denatured form has been developed and used for the parallel detection of specific antibodies in sera collected from women with abnormal PAP smears. The results show that this assay has an increased sensitivity compared to the tests based only on one viral protein, resulting in detection of a higher number of positive sera. The assay is not genotype-specific but it can be used to detect antibodies raised against genital HPVs other than type 16. This suggests that the 5 proteins belonging to different HPV genotypes share linear epitopes, as could be deduced from the high level of amino acid identity of these proteins. By comparing the amino acid sequences of the HPV16 L1, L2, E4, E6 and E7 to those of the other genotypes found in our study, the highest identity (>65%) is found among the L1 proteins. The homology among the E7 proteins is over 50%, similar to that of the E6 proteins, in contrast to L2, where homology is less than 50%. The sequence identity among the E4 proteins of related genotypes is less than 40%, and even less among distantly related genotypes (data not shown).

The specificity of the antigen-antibody interactions in the ELISA was indirectly measured by the avidity test, where immunoglobulins non-specifically bound to denatured proteins were removed by the urea treatment. As discussed above, most of the HPV-specific antibodies bound to the 5 antigens had AI higher than 40%, implicating specific antigen-antibody reactions, even in the sera of women infected with HPV other than type 16. These results suggest that the ELISA combined with the Avidity Index might improve the diagnostic value of serology survey in HPV infections.

Due to the limited number of sera tested in the present study, it is evident that this ELISA system needs validation in large-scale population studies, especially for the detection of HPV pan-reactive antibodies in people infected by genotypes different from HPV16. Nevertheless, in view of the known cross-reactivity between the most common HPV genotypes circulating in Europe [5,25], this ELISA should be useful in monitoring the pre-existing serostatus of women who receive HPV vaccination with the recently introduced prophylactic VLP-based vaccines.

In conclusion, the results of the ELISA introduced in this study are encouraging even though further investigations are needed to evaluate its usefulness in population screening for HPV infections. Moreover the assay is easy to set up since the recombinant proteins expressed and purified from *E. coli* are cost-effective and easy-to-standardize serological reagents [27-30]. The Histidine tail of these proteins allows a one-step purification procedure [31] and the purified proteins are readily applicable to novel platform technologies, which are useful when high numbers of samples need to be processed [32,33].

## Methods

### DNA constructs

The HPV16 genes were generated by PCR on pMHPV16d plasmid containing the complete virus genome (Accession number NCBI NC_001526) [34], kindly provided by A. Venuti, using specific primers carrying restriction sites useful for cloning in pQE-30 expression vector (QIAGEN), and were expressed as MRGS (H)_6 _tag proteins (Table 1). Cloning and expression of E7 (297 nt) were previously described by Accardi et al. 2005. A truncated form of L1 gene (1416 nt), was cloned into the restriction site SalI, the expressed protein starts at the second methionine of the L1 coding region and stops before the nuclear localization signal. The L2 (1422 nt), E6 (477 nt) and E4 (288 nt) genes were cloned between the restriction sites BamHI-HindIII of the vector. The plasmids were maintained in *E. coli *JM109 strain. The authenticity of the constructs was confirmed by DNA sequencing. The deduced amino acid sequences of the inserts were compared to those of the HPV16 (NC_001526), showing 100% identity.

### Protein expression, purification, SDS-PAGE, and Western-blot analysis

The recombinant proteins were purified using a denaturing protocol: bacterial cells transformed by L1, L2, E4, and E7 plasmids were lysed in a Phosphate buffer (100 mM Na_2_HPO_4 _300 mM NaCl, 10 mM Tris, 1% Triton X-100, pH 8), containing 8 M urea, while bacterial cells transformed by E6 plasmids were lysed in Phosphate buffer, containing 6 M Guanidine-HCl. Proteins were purified by affinity chromatography on Ni-NTA resin and eluted by a pH gradient. The fractions containing the proteins were pooled, adjusted to a neutral pH by adding Tris pH 8.8 and stored in urea buffer at -30°C, until use. Protein concentration was determined by standard method (BC protein assay, BIORAD). To evaluate the purity, each protein samples of L1, L2, E4, E6 and E7 were denatured in SDS-loading buffer (25 mM Tris-HCl pH 6.8, 5 % β-Mercapto-ethanol, 2% SDS, 50% glycerol), separated in pre-cast gel Nu-PAGE MES-SDS (INVITROGEN), stained by Gel Code Blue Stain Reagent (PIERCE). The proteins were identified by Western blot using the monoclonal anti poly-Histidine antibody Clone HIS1 (Sigma-Aldrich). A peroxidase-conjugate goat anti-mouse IgG (H+L) (SBA-INC USA) was used as secondary antibody. The immune-complexes were revealed by chemiluminescence (Amersham Bioscience).

### HPV genotype identification

HPV genotyping was performed by sequence analysis. DNA was extracted from the cells of cervical smears by QIAamp DNA mini kit (QIAGEN). To assess DNA integrity, the beta-globin gene was PCR amplified in each samples by the primers PCO3 and PCO4 [35]. To determine the presence of HPV DNA, each DNA sample was subjected to PCR amplification by the consensus primers GP5+/GP6+, which amplify a L1 region [36]. When the PCR result was negative and the serological test was positive, the DNA samples were further subjected to a semi-nested PCR using the MY11-GP6+ primers in the first round [37,38], and the GP5+/GP6+ couple of primers in the second round. Amplicons were detected by agarose gel electrophoresis, eluted by GFX PCR-DNA gel-band purification kit (Amersham Bioscience) and sequenced using the consensus primers GP5+/GP6+. The nucleotide sequences were aligned to the Gene-Bank Database sequences, using the BLAST program of the National Center for Biotechnology Information (NCBI) server [39]. A sequence identity of 98% was considered significant for the identification of the HPV genotype. The genotypes identified in the samples were HPV 6, 11, 16, 18, 31, 35, 39, 42, 51, 52, 53, 54, 56, 58, 66, 73, 81 and 90.

### Human sera and control sera

The sera analysed in this study were collected from women enrolled in the HPV-PathogenISS study. The women had an abnormal PAP smear as requested for the study design [11]. Only 99 women, of the 244 enrolled, gave the informed consent for serum taking. Serum samples were kept at -30°C, until used for analyses. Negative sera were randomly chosen among sera of individuals infected by other pathogens. As positive controls specific hyperimmune sera were used, obtained either in mice or in rabbits by injection of the recombinant purified HPV16 L1, L2, E4, E6 and E7 proteins. The use of hyperimmune anti-E7 polyclonal antibody has been reported before [40-42]. The immunisation protocol was as previously described in Di Bonito et al. [28].

### ELISA and avidity test

The denatured HPV16 recombinant proteins (0.25 μg/well) were adsorbed in carbonate buffer (pH 9.4) into Polysorp microtiter plates (NUNC) at 4° O/N. After a blocking step of 2 h at 37°C in phosphate buffer saline (PBS) containing 3% Non Fat Dry Milk (NFDM), the plates were incubated with 100 μl of human sera diluted 1:50 in 1% NFDM-PBS, at 37°C for 1 hr. The specific antigen-antibody complexes were detected by a peroxidase-conjugated goat anti-human IgG (H+L) (SBA INC. USA), using the TMB (VECTOR) as a substrate. After 30 min, the enzymatic reaction was stopped by adding 50 μl of 1 M sulphuric acid/well. Optical density was read at 450 nm. Washing steps were done with 200 μl/well of PBS containing 0.05 % Tween 20. The Monoclonal anti-poly-Histidine antibody was used as control of the coating step.

The avidity test was performed as described by Roque-Afonso et al. [25]. Serum samples were processed in parallel by a traditional ELISA and by an ELISA enclosing a 6 M urea-wash step. After incubation of serum samples with the antigens, the wells were washed three times (5 min each) with a PBS wash-buffer containing or not 6 M urea. Each reaction was done in duplicate. A final fourth PBS-wash was done in all wells. The Avidity Index (AI) was calculated as follows: AI = (absorbance reading with urea wash/absorbance reading without urea wash) × 100. The HPV- specific hyperimmune sera to L1, L2, E6, E7 and E4, the commercial anti-Histidine mAb (clone HIS-1, Sigma-Aldrich), anti- HPV16 L1 mAb (clone CamVir-1, Cymbus Biotechnology) and the anti-HPV16E7 mAb (clone 8C9, Zymed Laboratories INC) were used as controls.

### Cut-off definition and statistical analysis

To determine the cut-off values for each antigen, 40 control human sera were tested in ELISA against each of the five HPV recombinant proteins. The assay included the endpoint titre of the corresponding hyper-immune HPV sera as a positive control. The cut-off value was calculated as the arithmetic mean of the absorbance values of the negative sera, plus two standard deviation (SD). After the first cut-off calculation, the values of outlier serum samples were excluded, and the cut-off was re-calculated [43,44]. To control the reproducibility of the test, ten representative control human sera, the anti-poly-Histidine mAB and the hyperimmune serum specific to each HPV protein were always enclosed in each ELISA run. Each serum was assayed in duplicate and the mean of the absorbance value was taken as the final readout; the ELISA for each HPV protein was repeated three times. To evaluate the human serum variability, Coefficient of Variation (CV = SD/Mean × 100) was calculated [45]. All statistical tests were performed by Microsoft Office Excel software.

## Competing interests

The author(s) declare that they have no competing interests.

## Authors' contributions

PDB conceived the study, participated in acquisition, analysis and interpretation of data and in drafting the manuscript. FG, SM, MGD participated in acquisition, analysis and interpretation of data, LA participated in acquisition, analysis and interpretation of data and critically revised the manuscript, MB, SC; LM, AA, MC, participated in acquisition of the data; KS participated in design of the study and critically revised the manuscript. CG conceived the study, participated in its design, coordination, in acquisition of funding and helped to draft of the manuscript.

All authors have read and approved the final version of the manuscript.
